# Low fertility may be a significant determinant of ovarian cancer worldwide: an ecological analysis of cross- sectional data from 182 countries

**DOI:** 10.1186/s13048-018-0441-9

**Published:** 2018-08-16

**Authors:** Wenpeng You, Ian Symonds, Maciej Henneberg

**Affiliations:** 1 Adelaide Medical School, The University of Adelaide, Adelaide, SA Australia; 2 Institute of Evolutionary Medicine, University of Zürich, 8057 Zurich, Switzerland

**Keywords:** Ovarian cancer, Low fertility, Oxytocin, Significant predictor, Family well-being

## Abstract

**Background:**

Ageing, socioeconomic level, obesity, fertility, relaxed natural selection and urbanization have been postulated as the risk factors of ovarian cancer (OC56). We sought to identify which factor plays the most significant role in predicting OC56 incidence rate worldwide.

**Methods:**

Bivariate correlation analysis was performed to assess the relationships between country-specific estimates of ageing (measured by life expectancy), GDP PPP (Purchasing power parity), obesity prevalence, fertility (indexed by the crude birth rate), opportunity for natural selection (I_bs_) and urbanization. Partial correlation was used to compare contribution of different variables. Fisher A-to-Z was used to compare the correlation coefficients. Multiple linear regression (Enter and Stepwise) was conducted to identify significant determinants of OC56 incidence. ANOVA with post hoc Bonferroni analysis was performed to compare differences between the means of OC56 incidence rate and residuals of OC56 standardised on fertility and GDP respectively between the six WHO regions.

**Results:**

Bivariate analyses revealed that OC56 was significantly and strongly correlated to ageing, GDP, obesity, low fertility, I_bs_ and urbanization. However, partial correlation analysis identified that fertility and ageing were the only variables that had a significant correlation to OC56 incidence when the other five variables were kept statistically constant. Fisher A-to-Z revealed that OC56 had a significantly stronger correlation to low fertility than to ageing. Stepwise linear regression analysis only identified fertility as the significant predictor of OC56. ANOVA showed that, between the six WHO regions, multiple mean differences of OC56 incidence were significant, but all disappeared when the contributing effect of fertility on OC56 incidence rate was removed.

**Conclusions:**

Low fertility may be the most significant determining predictor of OC56 incidence worldwide.

**Electronic supplementary material:**

The online version of this article (10.1186/s13048-018-0441-9) contains supplementary material, which is available to authorized users.

## Background

Ovarian Cancer (OC56, abbreviated as per the International Classification of Diseases published by the WHO) [1] ranks among the top ten most commonly diagnosed cancers and top five deadliest cancers in most countries [2, 3]. In 2015, OC56 was present in 1.2 million women and resulted in 161,100 deaths worldwide [4] . In the twenty-first century, a woman’s overall lifetime risk of developing OC56 is around 1.6% [2, 5, 6], and her chance of dying of the disease is 1 in 100 [2, 6].

Although OC56 has been known to medical scientists for over 150 years [7], the aetiology of this lethal disease is not well understood. Most research on the aetiology of OC56 has focused on genetic and environmental carcinogenic factors, such as talc, pesticides, red meat and alcohol in diet, smoking, and herbicides. However, to date, none of these factors has been consistently shown to be a major risk factor for the development of OC56 [8]. Alternative hypotheses for the aetiology of the disease have also been suggested. Several studies have suggested that, obese women (those with a body mass index of at least 30 kg/m^2^) may have a greater risk of developing OC56 because of their elevated levels of circulating estrogen [9–11]. An accumulation of somatic mutations has been suggested as the mechanism for the higher incidence of the disease in women over the age of 45 [9]. Urbanization may have improved public hygiene, sanitation and access to health care for women [12], but it has been associated with public health issues, including OC56 [13] due to the changes in occupational, dietary and exercise patterns [6, 12, 14, 15].

Natural selection, as one of the key mechanisms of evolution, differentiates phenotypes’ survival and/or fertility that reflect genetic differences. The Biological State Index (I_bs_) has been constructed to measure the opportunity for natural selection through differential mortality at the population level. The I_bs_ calculation combines life table function d_x_ (number of deaths at age x) with the age-specific completed relative fertility rate s_x_ (fraction of total fertility rate to a woman up to age x): I_bs_ = 1 – Σd_x_s_x_ [16–21]. I_bs_ can be used as a way of measuring the opportunity for an individual born into a given population to pass on its genes to the next generation [17, 19, 22–24]. I_bs_ has been postulated to reflect changes in the mutation-selection balance as a result of the effect of improved healthcare on relaxing natural selection and thus measure the magnitude of accumulation of the deleterious genes [16], including those responsible for cancers such as OC56 [17], type 1 diabetes [18] and obesity [18, 19] in human populations.

The association between low fertility and OC56 risk has been well described and it has been postulated that this risk increases in women who have ovulated less over their lifetime either through infertility or administering the combined birth controls, such as contraceptive pills [6, 25–32].

To the best of our knowledge, despite that low fertility is a well-established risk factor for OC56, no research has compared the contributing effects of fertility to OC56 with other OC56 risk factors, such as ageing, I_bs_ (index of magnitude of OC56 genes accumulation in human populations), obesity and socioeconomic factors (GDP and urbanization).

There is significant variation in the incidence of OC56 between different geographic regions globally [2, 3, 33–35]. This phenomenon has also been observed in different populations [6, 13] within the same countries [36, 37]. A number of publications suggest that the disparity between regions and populations is related to socioeconomic level.

In this study, empirical macro-level data have been used to test the hypothesis that fertility (measured by the crude birth rate) is the principal determinant of developing OC56, and that it is fertility, instead of GDP, that is most important factor in shaping the regional variation of OC56 incidence rate.

## Methods

### Data sources

The following country specific data published by the agencies of the United Nations were analysed for this study.

1. The GLOBOCAN 2012 estimates of incidence rate of female OC56 [34].

GLOBOCAN provides contemporary population level estimates by cancer site and sex [2]. This project is conducted by the WHO research agency, the International Agency for Research on Cancer (IARC).

OC56 incidence rate is expressed as the number per 100,000 females who were diagnosed with OC56 in 2012. The age-standardized OC56 incidence rate was selected in the interest of the data comparability between countries.

2. The World Bank published data on crude birth rate, per capita GDP PPP and urbanization [38]

Crude birth rate (CBR) indicates the number of live births occurring during the year, per 1000 population estimated at midyear. CBR was used to index the fertility in this study over a 20 year period (1992) to reflect long exposure with delayed presentation of OC56. Terms “birth rate” and “fertility” are interchangeable in this paper.

Socio-economic level has been associated with OC56 risk [2, 34, 39, 40]. We chose per capita GDP purchasing power rate (GDP PPP in 2012 international $) because it takes into account the relative cost of local goods, services and inflation rates of the country.

Urbanization has been postulated as a major OC56 predictor [41, 42] because it represents the major demographic shift entailing lifestyle changes [12, 43, 44]. Urbanization is expressed with the country-specific percentage of total population living in urban areas in 2012.

3. The United Nations statistics division estimates of the life expectancy [45]

Country-specific life expectancy, which reflects ageing, has been well established to be correlated with OC56 incidence [46, 47]. Therefore, we selected life expectancy of older people (e_65_, 2005–2010) [45] to index the ageing process at population level.

4. The magnitude of OC56 gene accumulation in a population indexed with the biological state index (I_bs_)

The country specific I_bs_ was downloaded from the previous publication [19]. It has been postulated that reduced natural selection (measured by I_bs_) may have allowed accumulation of deleterious genes of non-communicable diseases [17–19], such as OC56 [17].

5. The WHO Global Health Observatory (GHO) data on obesity prevalence

Obese females may be at greater risk of developing OC56 than those who are not obese [48]. The country-specific percentage of the females aged 18+ with a BMI ≥ 30 kg/m^2^ in 2010 was extracted from the GHO data repository [49].

### Data selection

Country specific OC56 incidence rates, ageing, fertility, GDP, I_bs_, obesity and urbanization were collated for all countries where data were available. We extracted OC56 incidence rates for 182 countries and then the other variables were matched individually with OC56.

Each country was treated as an individual study subject in the data analysis. Not all the countries (subjects) had information for all the variables.

The relevant United Nations agencies offer free online access to data required for the analyses in this study. No ethics approval was required as there were no individual patients involved in the study.

### Data multicollinearity check

In order to avoid the inter-correlation between predictor variables, the multicollinearity statistics were calculated to test the correlations among the variables. Each variable was alternated as the dependent variable, and all the others were considered as the predictor variables in our analysis with the regression model. It was found that the collinearities between variables were not significant since only the tolerance of less than 0.20 and a VIF of more than 5 indicates a multicollinearity problem [50]. Values in our study were more than 0.20 and less than 5 respectively. Details are provided in Additional file 1.

### Data analysis

To assess the population level determinants of OC56, the analysis proceeded in five steps.

1. Scatter plots were produced with the original data in Microsoft Excel® to explore and visualize the strength, shape and direction of correlations of OC56 to fertility and GDP respectively.
2. Data were logarithmed to improve their homoscedasticity for linear regression analyses. Bivariate (Pearson’s r and nonparametric Spearman’s rho) correlations were performed to evaluate the direction and strength of the correlations between all the variables of all the subjects and effects possible effects of non-normality of distributions on the strength of moment-product correlations.
3. Partial correlation analysis of Pearson’s moment-product approach was performed. We alternated each of the six variables (ageing, fertility, GDP, I_bs_, obesity and urbanization) as the independent predictor when all other five variables were included as the potential confounding factors.
  Fisher’s r-to-z transformation was applied to assess the significance level of difference between pairs of correlation coefficients.
4. Standard multiple linear regression (enter) was performed to describe the correlations between the dependent variable (OC56) and the predicting variables. In order to explore if low fertility can partially explain why ageing, GDP, I_bs_, obesity and urbanization are correlated with OC56, the enter multiple linear regression was performed to determine the correlations between OC56 incidence and the risk factors in two models: (1) when fertility was incorporated; and (2) excluded as a predicting variable
  Subsequently, standard multiple linear regression (Stepwise) was performed to select the predicting variable (s) which have the greatest influence on OC56 in two versions: (1) when fertility was incorporated and (2) excluded as a predicting variable.
5. The equations of the best fitting non-linear trendlines displayed in the scatter plots analysis of relationships between OC56 incidence and fertility (y = 0.006 × ^2^–0.504× + 14.816, R^2^ = 0.485) and GDP PPP (y = 0.7167× + 0.2225, R^2^ = 0.2571) were used to calculate and remove the contributing effects of GDP PPP on OC56 incidence rate respectively by using regressions of OC56 residuals around fertility and GDP PPP. This allowed us to create two new dependent variables, “Residual of OC56 standardised on fertility” and “Residual of OC56 standardised on GDP PPP”

Means of the OC56 incidence rate, the “Residuals of OC56 standardised on fertility” and “Residuals of OC56 standardised on GDP PPP” of all the countries were calculated for mean difference comparisons.

Analysis of variance (ANOVA) was conducted to detect the significant differences among the means of OC56 incidence rate, “Residual of OC56 standardised on fertility” and “Residual of OC56 standardised on GDP PPP” between the six WHO regions [51]. Further post-hoc (Bonferroni) tests were performed to identify the source (pairs) of significant differences.

Bivariate correlations, multiple linear regression analysis (Enter and Stepwise) and ANOVA were conducted with SPSS v. 24. The raw data were used for calculation of mean OC56 incidence rate and “Residual of OC56 standardised on fertility” and “Residual of OC56 standardised on GDP PPP”. The significance was kept at the 0.05 level, but 0.01 and 0.001 levels were also reported. Standard multiple linear regression analysis criteria were set at probability of F to enter ≤0.05 and probability of F to remove ≥0.10.

## Results

**Fig. 1:**
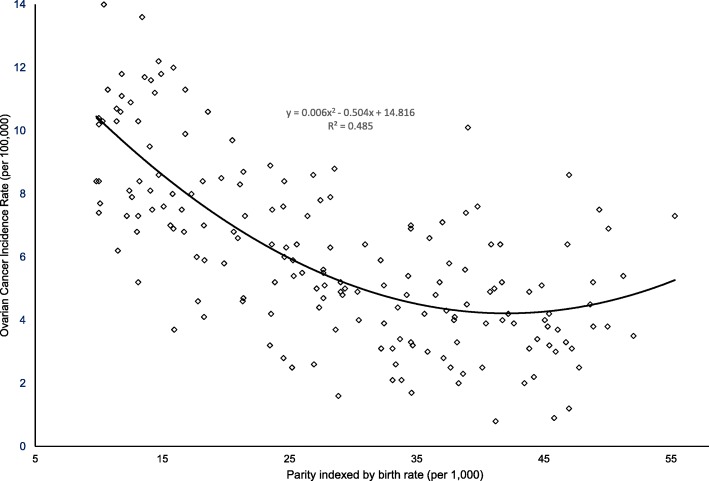
The relationship between fertility and ovarian cancer incidence rate

The relationship identified in the scatterplots between fertility and OC56 was noted to be polynomial with a strong, but inverse (negative) correlation (R^2^ = 0.485, *p* < 0.001, *n* = 179, Fig. 1).

The strong relationship between fertility and OC56 identified in the scatterplots was confirmed by the subsequent nonparametric and Pearson r analyses based on the log-transformed data.

Globally, fertility was significantly and negatively correlated to OC56 incidence (*r* = − 0.632 and rho = − 0.655, *p* < 0.001 respectively in Pearson and non-parametric analyses) (Table 1).

**Table 1:** Pearson r (above the diagonal) and nonparametric “rho” (below the diagonal) correlation between all variables

	OC56	Ageing	Fertility	GDP	Ibs	Obesity	Urbanization
OC56	1	0.394^***^	−0.632^***^	0.507^***^	0.455^***^	0.189^*^	0.280^***^
Ageing	0.428^***^	1	−0.737^***^	0.748^***^	0.766^***^	0.322^***^	0.570^***^
Fertility	−0.655^***^	− 0.769^***^	1	−0.772^***^	− 0.712^***^	− 0.338^***^	− 0.557^***^
GDP PPP	0.531^***^	0.759^***^	−0.813^***^	1	0.742^**^	0.485^***^	0.713^***^
I_bs_	0.602^***^	0.849^***^	−0.883^***^	0.858^***^	1	0.457^***^	0.551^***^
Obesity	0.169^*^	0.350^***^	− 0.377^***^	0.453^***^	0.409^***^	1	0.484^***^
Urbanization	0.345^***^	0.657^***^	−0.628^***^	0.781^***^	0.711^***^	0.506^***^	1

The table shows the bivariate correlation between all the variables. *** *p* < 0.001; Country number: 167–182

Ovarian cancer (OC56) incidence rate is from the International Agency for Research on Cancer. Birth rate indexing fertility, GDP PPP and urbanization are from the World Bank. Ageing expressed as life expectancy (e_65_) is from the United Nations. Obesity prevalence is from the World Health Organization

Biological State Index (I_bs_) was downloaded from previous publications, which were calculated with the data of the world fertility form the UN Population Council and the WHO life tables

It is also found that ageing, GDP, I_bs_, obesity and urbanization had strong and significant correlations to OC56 incidence in both Pearson and non-parametric analyses respectively (Table 1).

The relationship between OC56 and each independent variable (ageing, fertility, GDP, I_bs_, obesity and urbanization) was tested by keeping the other five variables statistically constant in partial correlation analysis. Fertility was the only predictor showing a substantial significant correlation (*r* = − 0.448, *p* < 0.001) with OC56 independent of the other five variables (Table 2). Ageing showed significant, but weak correlation to OC56 (*r* = − 0.178, *p* < 0.05). The Fisher r-to-z transformation revealed that OC56 was in significant stronger correlation with fertility than with ageing (z = 2.68, *p* < 0.01). GDP, I_bs_, obesity and urbanization showed significant correlation to OC56 in the bivariate correlation analyses respectively. However, none of these variables showed a significant correlation with OC56 independent of the other five predictors. This indicates that fertility is the only significant predictor of OC56 independent of the secondary association between OC56 incidence and I_bs_ (magnitude of OC56 accumulation) and environmental factors (ageing, fertility, GDP, obesity and urbanization).

**Table 2:** Comparison of partial correlation coefficients between ovarian cancer incidence and each variable when the other five variables are controlled for

Variables	Fertility			Ageing			GDP			I_bs_			Obesity			Urbanization		
	r	P	Df	R	p	df	r	p	df	r	p	df	r	p	df	r	p	df
Fertility	−0.448	< 0.001	160	–	–	–	–	–	–	–	–	–	–	–	–	–	–	–
Ageing	–	–	–	−0.178	0.023	160	–	–	–	–	–	–	–	–	–	–	–	–
GDP	–	–	–	–	–	–	0.148	0.060	160	–	–	–	–	–	–	–	–	–
I_bs_	–	–	–	–	–	–	–	–	–	0.079	0.315	160	–	–	–	–	–	–
Obesity	–	–	–	–	–	–	–	–	–	–	–	–	−0.048	0.544	160	–	–	–
Urbanization	–	–	–	–	–	–	–	–	–	–	–	–	–	–	–	−0.131	0.095	160

The table shows partial correlations between Ovarian cancer (OC56) incidence between each variable while the other four variables are controlled for. - Controlled variable

Ovarian cancer (OC56) incidence rate is from the International Agency for Research on Cancer. Fertility indexed by birth rate, GDP PPP and urbanization are from the World Bank. Ageing expressed as life expectancy (e_65_) is from the United Nations. Obesity prevalence is from the World Health Organization

Biological State Index (I_bs_) was downloaded from previous publications, which were calculated with the data of the world fertility form the UN Population Council and the WHO life tables

Standard multiple linear regression (enter) analysis was applied to predict OC56 incidence when ageing, fertility, GDP, obesity and urbanization were included as the independent predicting variables.

When fertility was excluded as one of the independent variables, GDP PPP (β = 0.471, *p* < 0.001) and I_bs_ (β = 0.250, *p* < 0.05) were the two significant variables related to OC56 incidence. However, when fertility was included as an independent predictor, only the correlation between fertility and OC56 incidence was strong and significant. None of the other five predictors showed strong and significant correlation to OC56 (Table 3). Similarly, in a stepwise linear regression model, when fertility was not included as one of the independent predictors, GDP and I_bs_ were selected as the variables having the greatest influence on the development of OC56. However, when fertility was included together with the other five independent variables, only fertility was selected as the most influential predictor of OC56 with the R^2^ increase from 0.278 to 0.434. This suggested that GDP and I_bs_ did not appear to account for the major part of the impact on OC56 incidence. This finding supports our previous suggestion that fertility is the significant predictor of OC56 incidence in partial correlation analysis.

**Table 3:** Independent predictors of ovarian cancer incidence rate based on multiple linear regression modelling (Enter)

Variable	β	Std. Error	Sig.	β	Std. Error	Sig.
Fertility	–	–	–	−0.694	0.111	< 0.001
Ageing	−0.037	0.341	0.752	−0.207	0.309	0.052
GDP	0.471	0.055	< 0.001	0.163	0.052	0.174
I_bs_	0.250	0.658	0.032	0.100	0.589	0.342
Overweight	−0.056	0.069	0.496	− 0.020	0.060	0.778
Urbanization	−0.122	0.105	0.211	−0.125	0.092	0.146

The table describes the multiple linear regression analysis (Enter) results including and excluding fertility as a predictor of breast cancer. df = 164; − excluded variable

Ovarian cancer (OC56) incidence rate is from the International Agency for Research on Cancer. Fertility indexed by birth rate, GDP PPP and urbanization are from the World Bank. Ageing expressed as life expectancy (e_65_) is from the United Nations. Obesity prevalence is from the World Health Organization

Biological State Index (I_bs_) was downloaded from previous publications, which were calculated with the data of the world fertility form the UN Population Council and the WHO life tables

Table 4 showed that the mean OC56 incidence rate was lowest in Africa (4.19) and highest in Europe (8.70). The means of OC56 in the other four regions were 5.89 (Americas), 5.19 (Eastern Mediterranean), 5.90 (South East Asia) and 6.63 (Western Pacific). A post hoc Bonferroni analysis conducted on the multiple comparisons of means revealed that there were a number of significant differences in mean OC56 incidence rates between different WHO regions (Table 4). Mean of OC56 incidence in Europe was significantly greater than in Africa, Americas, East Mediterranean, South East Asia and West Pacific. Mean of OC56 in Americas was significantly greater than in Africa. The regions with greater means of fertility had lower means of OC56 incidence rates (*r* = 0.985, *p* < 0.001, *n* = 6).

**Table 4:** Comparison of mean differences of fertility, residuals of ovarian cancer (OC56) standardised on fertility and GDP PPP respectively between WHO regions

OC56 incidence rate			Residual of OC56 standardised on fertility			Residual of OC56 standardised on GDP		
I n Mean	J	Mean difference (I-J)	I n Mean	J	Mean difference (I-J)	I n Mean	J	Mean difference (I-J)
AF *n* = 46 Mean = 4.19	AM	−1.70^*^	AF *n* = 45 Mean = − 0.29	AM	− 0.16	AF *n* = 44 Mean = − 0.15	AM	−0.03
	EM	−0.99		EM	−0.29		EM	1.03
	EU	−4.50^***^		EU	−0.30		EU	−2.15^***^
	SEA	−1.71		SEA	−0.67		SEA	−1.02
	WP	−2.44^**^		WP	−0.81		WP	−0.98
AM, *n* = 31 Mean = 5.89	AF	1.69^*^	AM n = 31 Mean = −0.13	AF	0.16	AM *n* = 29 Mean = −0.12	AF	0.03
	EM	0.70		EM	−0.13		EM	1.06
	EU	−2.81^***^		EU	−0.15		EU	−2.12^**^
	SEA	−0.01		SEA	−0.52		SEA	−0.99
	WP	−0.74		WP	−0.65		WP	−0.95
EM *n* = 22 Mean = 5.19	AF	0.99	EM *n* = 21 Mean = 0.001	AF	0.29	EM *n* = 18 Mean = −1.18	AF	−1.03
	AM	−0.70		AM	0.13		AM	−1.06
	EU	−3.51^***^		EU	−0.01		EU	−3.18^***^
	SEA	−0.71		SEA	−0.38		SEA	−2.05
	WP	−1.45		WP	−0.52		WP	−2.01
EU *n* = 50 Mean = 8.70	AF	4.50^***^	EU *n* = 49 Mean = 0.14	AF	0.30	EU n = 50 Mean = 2.00	AF	2.15^***^
	AM	2.81^***^		AM	0.15		AM	2.12^**^
	EM	3.51^***^		EM	0.01		EM	3.18^***^
	SEA	2.80^*^		SEA	−0.37		SEA	1.13
	WP	2.06^*^		WP	−0.50		WP	1.17
SEA *n* = 11 Mean = 5.90	AF	1.71	SEA n = 11 Mean = 0.38	AF	0.67	SEA *n* = 10 Mean = 0.87	AF	1.02
	AM	0.01		AM	0.52		AM	0.99
	EM	0.71		EM	0.38		EM	2.05
	EU	−2.80^*^		EU	0.37		EU	−1.13
	WP	−0.73		WP	−0.13		WP	0.04
WP n = 22 Mean = 6.63	AF	2.44^**^	WP n = 21 Mean = −0.01	AF	0.81	WP *n* = 19 Mean = 0.83	AF	0.98
	AM	0.74		AM	0.65		AM	0.95
	EM	1.45		EM	0.52		EM	2.01
	EU	−2.06^*^		EU	0.50		EU	−1.17
	SEA	0.73		SEA	0.13		SEA	−0.04

The mean difference comparison results conducted with One-way ANOVA Post hoc Bonferroni are reported. *p < 0.05, **p < 0.01, ***p < 0.001

Ovarian cancer (OC56) incidence rate is from the International Agency for Research on Cancer. Fertility indexed by birth rate, GDP PPP and urbanization are from the World Bank. Ageing expressed as life expectancy (e_65_) is from the United Nations. Obesity prevalence is from the World Health Organization

Biological State Index (I_bs_) was downloaded from previous publications, which were calculated with the data of the world fertility form the UN Population Council and the WHO life tables

A subsequent ANOVA with post hoc Bonferroni procedure performed on the means of “Residual of OC56 standardised on fertility” in different WHO regions showed there was no significant difference among and between regions (Table 4). Whilst the same procedure was performed on the means of “Residual of OC56 standardised on GDP PPP”, the developed region, Europe still had the significantly higher “Residual of OC56 standardised on GDP PPP” than Africa, Americas and East Mediterranean (Table 4). The results from the post hoc Bonferroni tests conducted on comparisons between the WHO regions suggested that regional variations of OC56 incidence may only reach statistically significant levels if the contributing effect of their respective fertility was included. In other words, except for fertility, the total contribution of the other OC56 risk factors to OC56 incidence may not be sufficient for the difference in mean rates to reach significance level. This result was supported by the findings identified in our previous partial correlation (Table 2) and multiple linear regression analyses (Table 3) that fertility is the critical risk factor for OC56.

## Discussion

The worldwide secular trend of increased OC56 incidence may have multiple etiologies, which may act through multiple mechanisms at different magnitudes. By examining the correlations of OC56 with low fertility, ageing, GDP, I_bs_, obesity and urbanization respectively, this study has shown that only fertility and aging were correlated with the OC56 incidence significantly, although the latter was not as strongly. Statistically, this may suggest that low fertility was the most significant risk factor for OC56 when compared to ageing, GDP, I_bs_, obesity and urbanization. This finding is in agreement with three studies conducted by Hankinson et al. [32], Vachon et al. [31] and Cramer et al. [52] respectively which concluded that fertility is a significantly greater predictor of OC56 risk than other commonly used epidemiological variables.

The relationship between female reproductive performance and gynecological cancers has been known for over 300 years [30, 53]. Previous studies in multiple different populations have shown that nulliparous women have a 30–60% greater risk than parous women [52, 54]. Studies also reported that each additional full-term pregnancy lowers OC56 risk by approximately 15% [54, 55]. The mechanism of the influence of childbearing on reducing OC56 risk may be that full-term pregnancy, post-partum period and sometimes the subsequent lactation involve anovulation, suppress secretion of pituitary gonadotropins, lower levels of oestrogen [56–59], lessen exposure of the ovaries to chronic inflammation and mutation [60], and reduce proliferation of malignant transformations in the inclusion cysts and clefts which are invaginated and formed in the ovarian epithelium during ovulation [61].

Recent studies suggested that women with greater fertility may receive the protection against developing OC56 because:

1. They may produce more oxytocin [62–69] due to positive interactions between family members, especially those between spouses [64, 65, 70, 71]. Oxytocin may inhibit the progression of human ovarian carcinoma cells [28, 29].
2. They may have less stress due to more positive psychological well-being from greater family size, reduces stress levels. This may make their neuroendocrine and immune systems more efficient to reduce the risk of cancer (developing OC56) [72–75].
3. They are more likely to seek health service and maintain a healthy lifestyle [76–79], which may have their developing OC56 diagnosed earlier and removed in time.

This study revealed that low fertility determines the variation of OC56 incidence rate among the WHO regions. This finding contradicts the WHO and IARC’s statement that socioeconomic level is the determinant of regional variation of OC56 incidence rate [2, 6, 34]. This may suggest that the correlation between fertility and socioeconomic status (SES) is spurious – caused by the correlation of both variables (SES and OC56 incidence) to the same one (fertility) [80–82].

The strength of this study is that it uses an ecological study approach, different from hitherto used approaches, to demonstrate that low fertility is a significant determinant of OC56 risk.

We need to note several limitations of this study:

1. Each country was considered as a whole subject for the ecological study. The country-specific data included in this study may be different from those collected from individual participants. Therefore, the correlations identified from the data analysis may not hold true for all the individuals to have the risk in OC56 development.
2. There may be some random errors that occurred when the United Nations and its agencies collected and aggregated data at country level. Data from developed countries may be more complete than those from developing countries.
3. There are different categories of OC56, but we could not differentiate them for the correlation analysis due to the unavailability of such data.

## Conclusion

Low fertility appears to be a significant and strong determinant of OC56 risk independent of ageing, GDP, I_bs_, obesity and urbanization. These findings may be helpful for governments, policy-makers, funders, clinicians and researchers when determining future screening and primary presentation strategies for the disease [32, 83, 84].

## Additional file

Additional file 1: AF 1 Collinearity among the variables. (DOCX 16 kb)

## Abbreviations

FAO: The Food and Agriculture Organization of the United Nations
GDP PPP: Gross domestic product purchasing power parity
IARC: International Agency for Research on Cancer
I_bs_: Biological State Index
OC56: Ovarian Cancer
UN: The United Nations
WHO: World Health Organization
