# Supplementary material for: Low fertility may be a significant determinant of ovarian cancer worldwide: an ecological analysis of cross- sectional data from 182 countries
Source: J Ovarian Res. 2018 Aug 16;11:68. doi: 10.1186/s13048-018-0441-9 (PMC6097201; doi:10.1186/s13048-018-0441-9)
Supplement: Supplementary file 1 — AF 1 Collinearity among the variables. (DOCX 16 kb) [file 13048_2018_441_MOESM1_ESM.docx]

| Coefficients^a^ | | | |
| --- | --- | --- | --- |
| Model | | Collinearity Statistics | |
|  |  | Tolerance | VIF |
| 1 | Ageing e(65) UN LN | .305 | 3.276 |
|  | Birth rate 1992 LN | .337 | 2.967 |
|  | GDP PPP 2012 LN | .240 | 4.169 |
|  | Ibs LN | .313 | 3.192 |
|  | Obesity asr (%) ln | .654 | 1.529 |
|  | Urban 2012 LN | .465 | 2.149 |
| a. Dependent Variable: OCa rate asr LN | | | |

| Coefficients^a^ | | | |
| --- | --- | --- | --- |
| Model | | Collinearity Statistics | |
|  |  | Tolerance | VIF |
| 1 | Birth rate 1992 LN | .283 | 3.530 |
|  | GDP PPP 2012 LN | .251 | 3.991 |
|  | Ibs LN | .388 | 2.578 |
|  | Obesity asr (%) ln | .677 | 1.477 |
|  | Urban 2012 LN | .461 | 2.167 |
|  | OCa rate asr LN | .553 | 1.810 |
| a. Dependent Variable: Ageing e(65) UN LN | | | |

| Coefficients^a^ | | | |
| --- | --- | --- | --- |
| Model | | Collinearity Statistics | |
|  |  | Tolerance | VIF |
| 1 | GDP PPP 2012 LN | .257 | 3.892 |
|  | Ibs LN | .317 | 3.157 |
|  | Obesity asr (%) ln | .656 | 1.526 |
|  | Urban 2012 LN | .461 | 2.171 |
|  | OCa rate asr LN | .702 | 1.425 |
|  | Ageing e(65) UN LN | .326 | 3.069 |
| a. Dependent Variable: Birth rate 1992 LN | | | |

| Coefficients^a^ | | | |
| --- | --- | --- | --- |
| Model | | Collinearity Statistics | |
|  |  | Tolerance | VIF |
| 1 | Ibs LN | .321 | 3.112 |
|  | Obesity asr (%) ln | .676 | 1.479 |
|  | Urban 2012 LN | .544 | 1.839 |
|  | OCa rate asr LN | .546 | 1.832 |
|  | Ageing e(65) UN LN | .315 | 3.174 |
|  | Birth rate 1992 LN | .281 | 3.561 |
| a. Dependent Variable: GDP PPP 2012 LN | | | |

| Coefficients^a^ | | | |
| --- | --- | --- | --- |
| Model | | Collinearity Statistics | |
|  |  | Tolerance | VIF |
| 1 | Obesity asr (%) ln | .693 | 1.442 |
|  | Urban 2012 LN | .459 | 2.177 |
|  | OCa rate asr LN | .542 | 1.843 |
|  | Ageing e(65) UN LN | .371 | 2.695 |
|  | Birth rate 1992 LN | .263 | 3.795 |
|  | GDP PPP 2012 LN | .245 | 4.089 |
| a. Dependent Variable: Ibs LN | | | |

| Coefficients^a^ | | | |
| --- | --- | --- | --- |
| Model | | Collinearity Statistics | |
|  |  | Tolerance | VIF |
| 1 | Urban 2012 LN | .493 | 2.030 |
|  | OCa rate asr LN | .540 | 1.853 |
|  | Ageing e(65) UN LN | .309 | 3.240 |
|  | Birth rate 1992 LN | .260 | 3.849 |
|  | GDP PPP 2012 LN | .245 | 4.080 |
|  | Ibs LN | .330 | 3.028 |
| a. Dependent Variable: Obesity asr (%) ln | | | |

| Coefficients^a^ | | | |
| --- | --- | --- | --- |
| Model | | Collinearity Statistics | |
|  |  | Tolerance | VIF |
| 1 | OCa rate asr LN | .547 | 1.829 |
|  | Ageing e(65) UN LN | .300 | 3.338 |
|  | Birth rate 1992 LN | .260 | 3.846 |
|  | GDP PPP 2012 LN | .281 | 3.561 |
|  | Ibs LN | .312 | 3.207 |
|  | Obesity asr (%) ln | .702 | 1.425 |
| a. Dependent Variable: Urban 2012 LN | | | |
